# One for All? Hitting Multiple Alzheimer's Disease Targets with One Drug

**DOI:** 10.3389/fnins.2016.00177

**Published:** 2016-04-25

**Authors:** Rebecca E. Hughes, Katarina Nikolic, Rona R. Ramsay

**Affiliations:** ^1^School of Biology, BMS Building, University of St AndrewsSt Andrews, UK; ^2^Department of Pharmaceutical Chemistry, Faculty of Pharmacy, University of BelgradeBelgrade, Serbia

**Keywords:** multi-target drugs, Alzheimer's Disease, *in silico*, datamining, rational drug design, repurposing

## Abstract

**HIGHLIGHTS**

- Many AD target combinations are being explored for multi-target drug design.
- New databases and models increase the potential of computational drug design
- Liraglutide and other antidiabetics are strong candidates for repurposing to AD.
- Donecopride a dual 5-HT/AChE inhibitor shows promise in pre-clinical studies

Alzheimer's Disease is a complex and multifactorial disease for which the mechanism is still not fully understood. As new insights into disease progression are discovered, new drugs must be designed to target those aspects of the disease that cause neuronal damage rather than just the symptoms currently addressed by single target drugs. It is becoming possible to target several aspects of the disease pathology at once using multi-target drugs (MTDs). Intended as an introduction for non-experts, this review describes the key MTD design approaches, namely structure-based, *in silico*, and data-mining, to evaluate what is preventing compounds progressing through the clinic to the market. Repurposing current drugs using their off-target effects reduces the cost of development, time to launch, and the uncertainty associated with safety and pharmacokinetics. The most promising drugs currently being investigated for repurposing to Alzheimer's Disease are rasagiline, originally developed for the treatment of Parkinson's Disease, and liraglutide, an antidiabetic. Rational drug design can combine pharmacophores of multiple drugs, systematically change functional groups, and rank them by virtual screening. Hits confirmed experimentally are rationally modified to generate an effective multi-potent lead compound. Examples from this approach are ASS234 with properties similar to rasagiline, and donecopride, a hybrid of an acetylcholinesterase inhibitor and a 5-HT_4_ receptor agonist with pro-cognitive effects. Exploiting these interdisciplinary approaches, public-private collaborative lead factories promise faster delivery of new drugs to the clinic.

## Introduction

Current therapies available for the treatment of Alzheimer's Disease (AD) show limited ability to modify the disease. To date the focus for AD has been on the depletion of acetylcholine, but AD is a complex multifactorial disease with diverse clinical symptoms. AD causes the gradual onset of multiple cognitive deficits, affecting language, episodic memory and attention (Karran et al., 2011). The disease pathology includes extracellular amyloid beta (Aβ) plaques, intracellular neurofibrillary tangles, inflammation, oxidative stress, iron dysregulation and ultimately neuronal cell death (Carreiras et al., 2013). It is accepted that multifactorial diseases require the simultaneous modulation of multiple targets to manage the course of disease progression (Cavalli et al., 2008), leading to growth in multi-target drug (MTD) design (Hopkins, 2008). This review focuses on possible targets, their combinations, and the three main approaches to designing MTDs: structure-based design, data mining/repurposing, and *in silico* screening. The application of rational drug design to MTDs is difficult but with the recent advances in experimental and computational approaches, a combined approach harnessing the best attributes of each method should ultimately deliver success in the future.

## Targets and current therapies; limitations in alzheimer's disease

**Figure 1:**
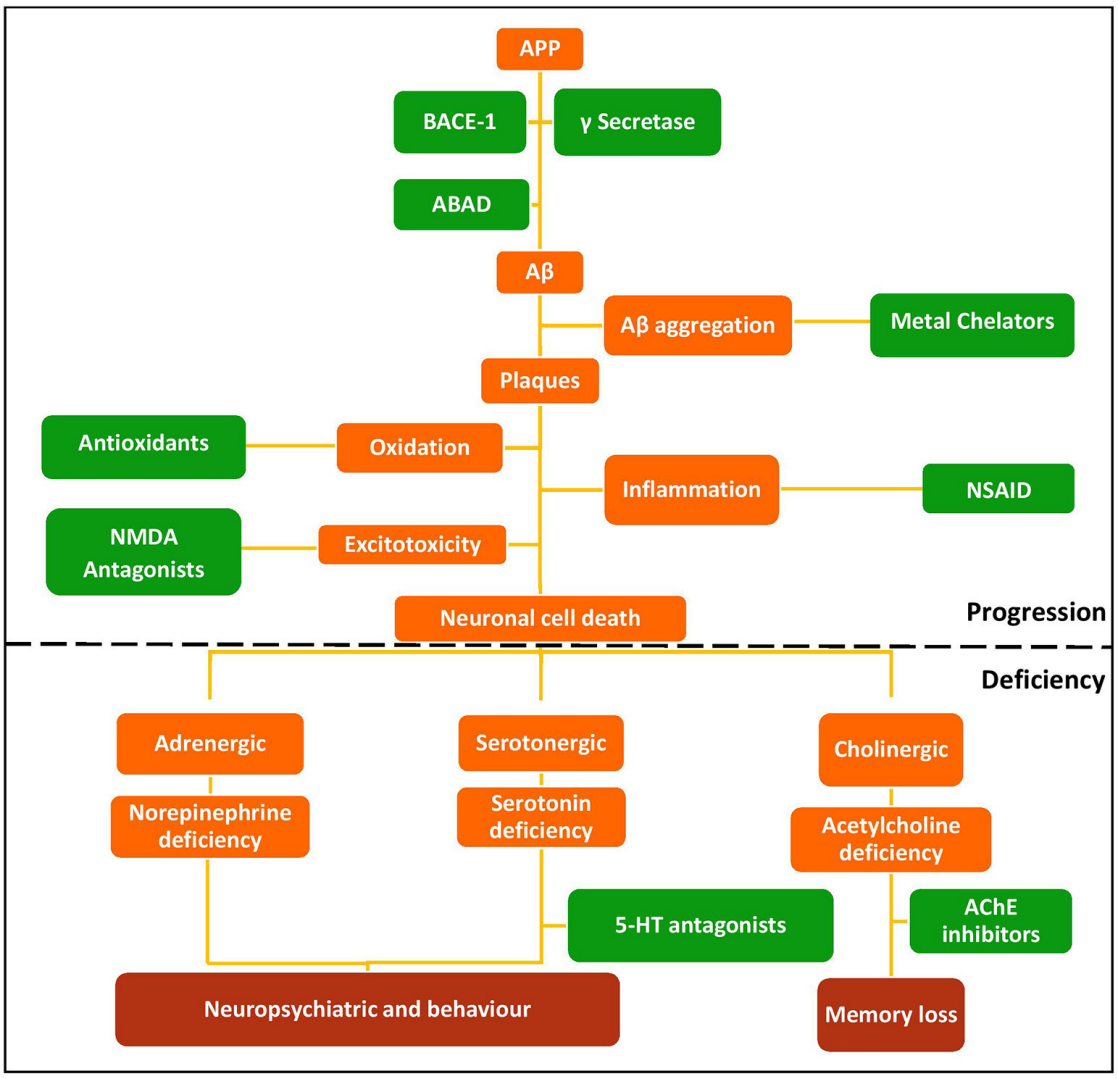
**Schematic of the amyloid hypothesis, the targets associated with AD, and potential drug intervention strategies**. Red, disease outcomes; Orange, Pathology; Green, Intervention strategies and drug classes.

The multifactorial nature of AD means there are many possible therapeutic targets. Current monotherapeutic treatments focus mainly on acetylcholinesterase (AChE) inhibition due to the early cholinergic hypothesis that cognitive dysfunctions of AD may be attributed to decreased neurotransmission at cholinergic synapses as a result of neuronal cell death (Bartus et al., 1982). AChE therapies provide symptomatic relief, but fail to reverse disease progression (Wilkinson et al., 2004; Deardorff et al., 2015), although recent work suggests that donepezil (Table 1) may enhance Aβ clearance (Mohamed et al., 2015). The amyloid hypothesis that the generation of toxic Aβ from amyloid precursor protein (APP) and Aβ aggregation result in the pathophysiological changes associated with AD (Figure 1) led to compounds targeting Aβ (Karran et al., 2011; Eisele et al., 2015). Although small molecules can target Aβ aggregation (see Table 1), the main strategy is immunotherapy, still in clinical trials (Palmer, 2011; Wisniewski and Goñi, 2014). Better understanding of protein aggregation is now available to guide therapeutic intervention on both Aβ and tau aggregation (Eisele et al., 2015).

**Table 1:** **Targets, drugs, and new multi-target ligands to combat Alzheimer's Disease (AD)**.

**Targets**	**Drug name**	**Structure**	**Comment**
**(A) APPROVED DRUGS**			
Acetylcholinesterase	Donepezil	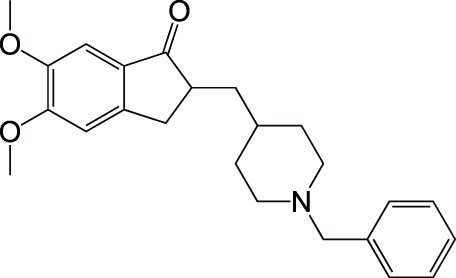	Approved drug
	Rivastigmine	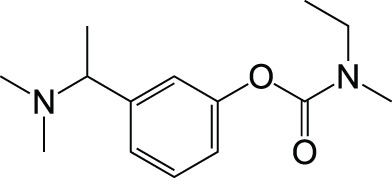	Approved drug
	Galantamine	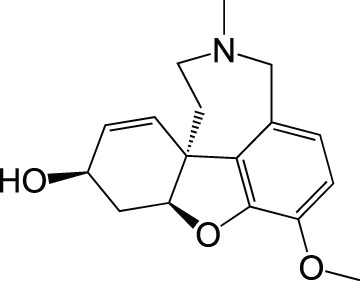	Approved drug
	Tacrine	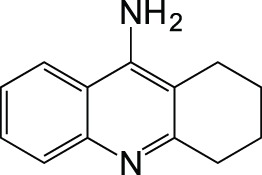	Withdrawn
Monoamine oxidase	Rasagiline	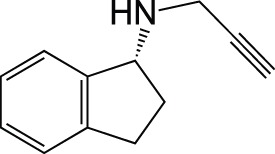	Approved for PD Phase II for AD
**Targets**	**Parent compounds**	**Compound**	**References**
**(B) MULTI-TARGET DESIGNED LIGANDS**			
Cholinesterase Aβ aggregation	Oxoisoaporphine Tacrine	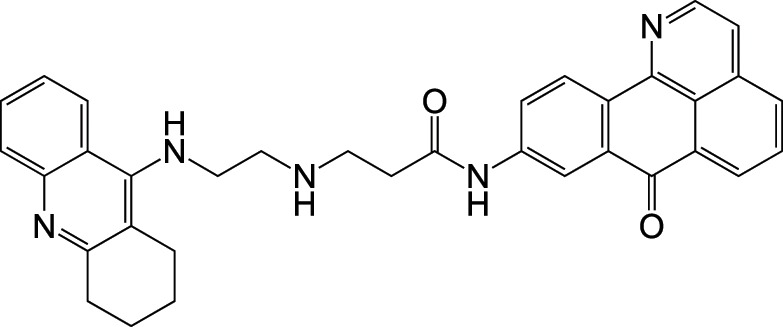	Tang et al., 2011
Cholinesterase Aβ aggregation	Donepezil	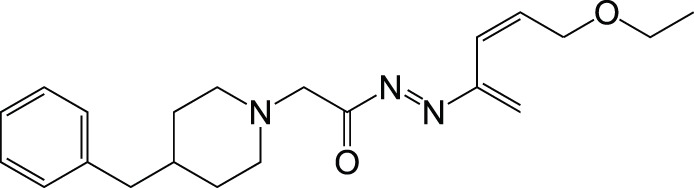	Ozer et al., 2013
AChE BACE-1	Huprine Tacrine	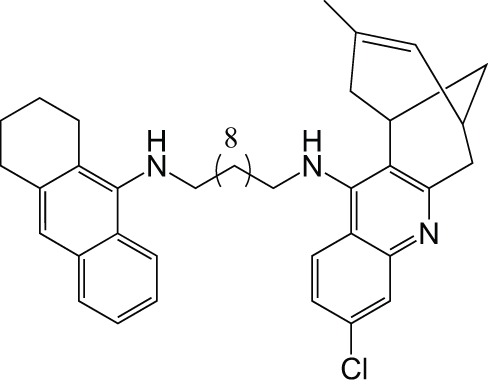	Galdeano et al., 2012
Cholinesterase MAO Aβ aggregation	Donepezil Compound 28 (Pérez et al., 1999)	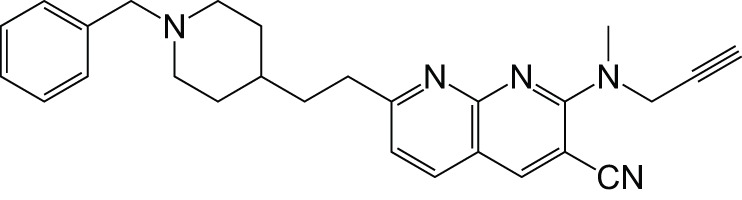	Samadi et al., 2012
Cholinesterase MAO	Donepezil Compound 28	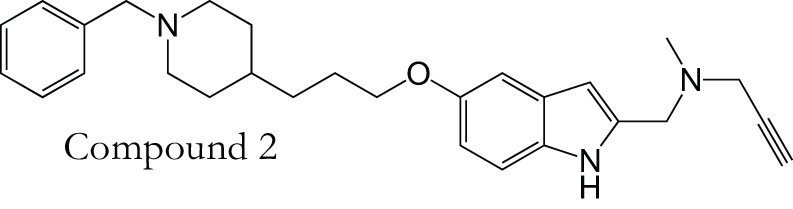	Bolea et al., 2011
MAO Cholinesterase	Donepezil PF9601N	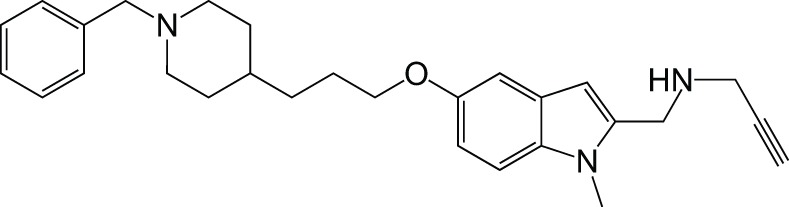	Bautista-Aguilera et al., 2014b
MAO Cholinesterase	Tacrine Selegiline (deprenyl)	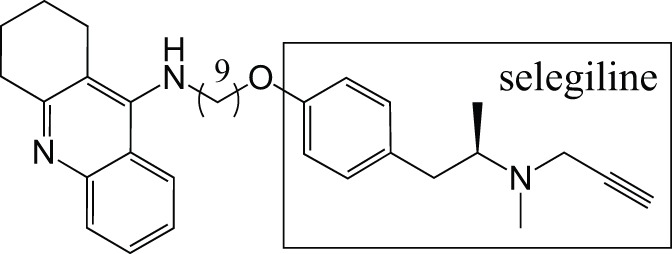	Lu et al., 2013b
Cholinesterase Reactive oxygen species	Memoquin Lipoic acid	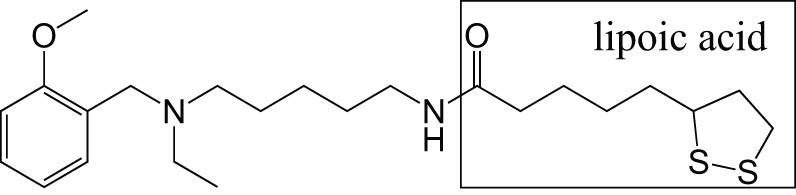	Rosini et al., 2011
MAO Metal chelator Aβ aggregation Cholinesterase	Resveratrol	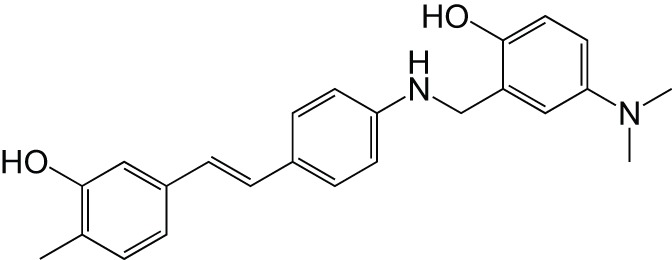	Lu et al., 2013a
Metal chelator Aβ aggregation	Resveratrol Clioquinol	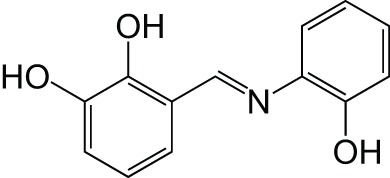	Li et al., 2014

Following on from direct targeting of Aβ, a relatively new target in AD is the β-secretase (BACE-1) enzyme. Inhibition of this protease BACE-1 should reduce the levels of Aβ in the brain (Vassar, 2014). BACE-1 inhibitors in clinical trials include AZD3293 in phase II/III (NCT02245737) and MK8931 (verubecestat) in phase III (NCT01953601). While these drugs show promise, there are potential complications from a possible role in synaptic function (Kandalepas and Vassar, 2014) and increased memory impairment and seizures in BACE-1 –/– mice (Hu et al., 2016).

Another major enzyme target, monoamine oxidase (MAO) is inhibited in Parkinson's Disease (PD) to spare the neurotransmitters depleted by neuronal death. The PD drug, rasagiline (see below), is a MAO-B inhibitor in clinical trials for AD (Weinreb et al., 2010).

N-methyl-D-aspartate (NMDA) is a glutamate receptor and is the only other target besides AChE for which a therapy has been approved (Anand et al., 2014). NMDA receptors are over-stimulated in AD brains due to excessive release of glutamate from neurons. This causes high levels of calcium ion influx and activation of enzymes that can cause neuronal cell death (Zheng et al., 2014). NMDA receptors have also been linked to tau hyper-phosphorylation and Aβ toxicity (Couratier et al., 1996). Memantine is the only approved NMDA receptor antagonist for the treatment of moderate to severe AD (Lipton, 2006).

Other receptors being explored are the 5-hydroxytryptamine (5-HT) serotonin receptors, in particular subtype 4 (5-HT_4_R) that is involved in mood, memory, and learning. 5-HT_4_R has been linked to memory deficits such as those seen in AD (Cho and Hu, 2007; Lezoualc'h, 2007; Russo et al., 2009). Stimulation causes release of ACh and increases other neurotransmitters (Licht et al., 2010).

Inhibition of these targets individually with current drugs (Table 1) has been ineffective at reversing the progression of the disease. A possible answer lies in a polypharmacological approach to modify activities of several of these targets simultaneously, particularly those associated with the progression of the disease. MTDs developed for AD have modified two or more of known targets (cholinesterases, MAOs, acetylcholine receptors, serotonin receptors) or have properties thought to retard disease progression, such as metal chelation, antioxidant or anti-inflammatory activity, or prevention of Aβ or tau aggregation. Ligands for combinations of drug targets should be evaluated against disease progression to define optimal target combinations.

## Datamining and repurposing

Repurposing is the development of existing or abandoned drugs for new indications (Boguski et al., 2009), related to the original purpose or after off-target effects are identified by datamining. Repurposing reduces the time to launch, cost of development, and the uncertainty associated with safety and pharmacokinetics (Kim, 2015).

Datamining is a way of using pre-existing knowledge about molecules and applying it to develop new drugs (Sirota et al., 2011; Corbett et al., 2012, 2013). For example, clinical data can be used to identify unanticipated benefits in side effects seen in clinical trials, allowing the early repurposing of therapies (Loging et al., 2011). The most promising drug currently being investigated for repurposing is rasagiline, a selective, irreversible MAO-B inhibitor for the treatment of PD (Youdim et al., 1995, 2001). The repurposing to AD stems from the ability of rasagiline to regulate the non-amyloidogenic processing of APP (Yogev-Falach et al., 2003; Bar-Am et al., 2004). Rasagiline also shows neuroprotective activity due to the propargylamine moiety that activates Bcl-2 and down-regulates the Bax proteins (Youdim et al., 2005). It is now in Phase II trial for the treatment of AD (www.clinicaltrials.gov/ct2/show/NCT02359552).

Natural products provide another potential source of MTDs (Ji et al., 2009; Prati et al., 2014). Datamining could harness their potential and then synthetic analogs and derivatives created to enhance their activity (Agis-Torres et al., 2014). Huperzine A, a dietary supplement in China, is an AChE inhibitor (Xing et al., 2014) seemingly with beneficial effects on cognitive function and daily living in AD patients, but the clinical trials to date have poor methodological approaches and so results are inconclusive (Xu et al., 1995; Rafii et al., 2011). Synthetic derivatives like huprine X and huprine-tacrine hybrids have been developed to improve on the potency and efficacy of huperzine A (Galdeano et al., 2012).

One promising repurposing area is from diabetes to AD, first considered because type 2 diabetes is a risk factor for AD (Schrijvers et al., 2010). Insulin signaling is impaired in the brains of Alzheimer's patients (Moloney et al., 2010) so several aspects of insulin signaling and regulation have been targeted. For example, Phase II clinical trials of intranasal insulin administration showed improved biomarkers for early AD including amyloid and tau indicators in cerebrospinal fluid (CSF) (Craft et al., 2012). Long-lasting glucagon-like peptide 1 (GLP-1) analogs that promote the secretion of insulin are also in clinical trials. Three GLP-1 analogs with potential therapeutic value in AD (Corbett et al., 2012; Hölscher, 2014), have shown *in vivo* benefits in mouse AD models (Gengler et al., 2012). Two of these are Exendin-4 in Phase II clinical trial (See www.clinicaltrials.gov/ct/show/NCT01255163) and Liraglutide, a GLP-1 receptor agonist. Liraglutide crosses the blood brain barrier (BBB) in animal models (Hunter and Hölscher, 2012). It decreases intracellular APP, Aβ, and Fe^2+^-related neurodegeneration, but also improves synaptic plasticity and cognitive function, reducing AD pathology (McClean et al., 2011; McClean and Hölscher, 2014).

## Databases and open-access mining

The construction of AD knowledge bases will facilitate the repurposing of drugs and our understanding of the neurosignaling pathways involved. One such example is AlzPlatform, an AD domain-specific chemogenomics database allowing the identification of off-target effects and the repurposing of compounds (Liu et al., 2014). AlzPlatform contains the established computational algorithm TargetHunter, which is an *in silico* target identification tool for small molecules (Wang et al., 2013).

While there is a wealth of open access chemical information available to aid repurposing of FDA approved drugs, compound libraries held by pharmaceutical companies are, for the most part, unavailable. Datamining to find possible targets for these compounds could prove fruitful. An open-innovation drug-repositioning project between AstraZeneca and the Medical Research Council (http://www.labtalk.astrazeneca.com/hot-topics/open-innovation-in-drug-repositioning/) has started and the National Center for Advancing Translational Sciences (NCATS) has created a similar collaboration with 8 pharmaceutical companies. One of the compounds from this program is the Src tyrosine kinase inhibitor, AZD0530 (Saracatinib), being repurposed for the treatment of AD (Larson et al., 2012; Nygaard et al., 2014). The European Lead Factory (www.europeanleadfactory.eu) is another initiative set up to allow sharing of commercial compound libraries via collaborative public-private partnerships. The compound library, a pool of 30 partner libraries, provides a collection of over 500,000 compounds available for academic groups and pharmaceutical companies to screen experimentally against their chosen targets with great potential to generate new therapeutics.

## Rational drug design

Rational drug design is a traditional method for drug development based on structure-function analysis. Successful rational drug design requires the logical and systematic changing of substituents to modify the properties of a compound to reach the desired effect. When applied to MTD design, it means combining pharmacophores of multiple drugs to give complex combinations, then compensating for any disadvantage to the individual targets.

M30, a brain-permeable iron chelator and brain selective MAO inhibitor, was designed on the rationale that MAO and iron are elevated in the brains of AD patients, and this leads to oxidative stress and neurodegeneration (Youdim, 2006). The MAO inhibition is due to the propargylamine of the FDA-approved anti-PD drug rasagiline (Youdim, 2013). The iron chelating activity comes from the prototype iron chelator VK28 (Zheng et al., 2014). M30 irreversibly inhibits both MAO-A and -B with IC_50_ values of 37 ± 2 nM and 57 ± 1 nM respectively, less selective than rasagiline (412 nM and 4.4 nM, respectively) (Zheng et al., 2005). It also regulates APP via its iron chelating ability since APP is a metalloprotein with an iron responsive element in the 5′ untranslated region (UTR) (Youdim, 2013). Therefore, it has a direct effect on Aβ levels.

To reduce the possibility of adverse effects of metal chelation in the periphery, a second-generation molecule and prodrug, M30D, was designed from tacrine, rivastigmine, and rasagiline (Zheng et al., 2010a,b, 2014). It was 3-fold more potent against MAO-A (IC_50_ of 7.7 nM) than the parental molecule, retaining the same MAO-B inhibition (Zheng et al., 2014). It is metabolized to the active chelator form M30 by AChE and inhibits AChE with an IC_50_ of 500 nM in rat brain homogenates. With these characteristics, M30D is the first site-directed metal chelator with the potential to treat AD.

A similar MTD is ASS234 composed from donepezil and the propargylamine PF9601N. It binds to all the AChE/BuChE and MAO A/B enzymes, shows antioxidant, neuroprotective and suitable permeability properties. *In vivo*, ASS234 restores scopolamine-induced cognitive impairment to the same extent as donepezil, is less toxic, and prevents β-amyloid aggregation in the cortex of AD transgenic mice (Bolea et al., 2013; del Pino et al., 2014).

Another promising structure-based MTD is donecopride. Donecopride is a structural hybrid of donepezil, an AChE inhibitor, and RS67333, a 5-HT_4_R agonist. RS67333 is a partial 5-HT_4_R agonist that exerts a procognitive effect via its ability to promote cleavage of APP. RS67333 is structurally similar to donepezil, so it was postulated that it might inhibit AChE. It was indeed a submicromolar AChE inhibitor (Lecoutey et al., 2014; Rochais et al., 2015). Derivatives of RS67333 were then synthesized to improve the AChE activity, from which MR31147 (donecopride) was selected for its remarkable multi-target activity *in vitro*, including an IC50 for AChE of 16 nM (Lecoutey et al., 2014). Donecopride crosses BBB, has a nontoxic profile, and exerts a procognitive effect *in vivo* (Lecoutey et al., 2014). It will be interesting to see how this promising molecule progresses since 5-HT_4_ targeted therapies have been less commonly explored for AD.

These examples show how methodical rational drug design and experimental screening can be used to generate promising MTD candidates for the treatment of AD.

## *In silico* screening

Rational drug design is currently the approach of choice for MTD design but it is labor intensive, so there is a need for more *in silico* screening to reduce costs and accelerate progress. New computer-based approaches for *in silico* screening (Ekins et al., 2007a; Wang et al., 2013), such as quantitative structure-activity relationships, molecular modeling approaches, machine learning, data mining, and data analysis tools, use *in vitro* data to generate predictive models. Such models are very useful in the discovery and optimization of novel ligands with enhanced affinity to a drug target, as well as for optimization of physicochemical and pharmacokinetic properties of the drug candidates. Several of these *in silico* methods will be evaluated here in the context of selected MTD examples.

Virtual ligand screening is an alternative to experimental high throughput screening (Ekins et al., 2007a). It can be used to screen whole databases of molecules and rank them based on their probability of binding to a drug target. The highest-ranking molecules can then be taken through to an experimental stage to confirm the hits and determine the most promising leads. Virtual screening can be either ligand based, where a diverse set of ligands are analyzed to build up a pharmacophore to score the screen against, or it can be structure-based, where molecules are docked onto the target and scored based on their likely affinity for the target. Quantitative structure-activity relationships (QSAR) is a form of ligand-based virtual screening but it uses a series of logic-based rules describing the chemical properties and substructures linked to activity to screen a database of molecules (Ekins et al., 2007b).

Recently a virtual ligand screen (Bautista-Aguilera et al., 2014a) was used for lead optimization of two donepezil hybrid compounds shown previously to inhibit MAO-A, MAO-B, AChE, and BuChE (Bolea et al., 2011). The 3D-QSAR analysis was carried out both to explain the binding of these compounds to the active sites of the enzymes and to predict substitutions that would increase binding. The QSAR model was used to generate 19 new molecules with substitutions predicted to increase the activity against MAOs and ChEs. The predicted IC50 values suggested that N-[(5-benzyloxy-1-methyl-1-H-indol-2-yl)methyl]-N-methylprop-2-yn-1-amine (compound 2) would be the most potent inhibitor of all four enzymes with values in the nanomolar range for MAO-A, MAO-B, and AChE, while BuChE was predicted at 1.3 μM. The experimental results showed that only 7 of the 19 compounds were active against all four enzymes, which demonstrates how difficult it can be to get activity against four enzymes in one molecule. However, as predicted, compound 2 was the most potent against all four enzymes, confirming the quality of the 3D-pharmacophores. Thus, *in silico* screening can be used to modify a lead compound and generate effective multi-potent inhibitors (Bautista-Aguilera et al., 2014a).

Another option, rather than start from a lead molecule, is to use pharmacophore models to screen existing compound databases with the aim of generating hits against multiple targets. This approach has been used to identify dual inhibitors of BACE-1 and AChE using a combination of virtual screening and molecular docking of compounds from three compound databases. The strategy adopted follows a sequential screening model where the databases were screened in parallel against the two targets sequentially before filters were applied to select for compounds with desirable properties. Finally, docking of the chosen compounds led to 8 compounds from the original 501,799 that fit all the given criteria (Tyagi et al., 2010).

Importantly, *in silico* screening allows the early filtering of compounds based on properties, such as BBB permeability, which can reduce late stage attrition. However, screening sequentially means that compounds that fall below the cut-off criteria in the first round are not screened against the second target. To avoid loss of potential candidates, screening against the targets in parallel is essential (Steindl et al., 2006).

## Concluding remarks

Over the past decade, there has been a substantial research effort to design MTDs for the treatment of AD. This focus on MTDs is aided by the understanding that AD is a complex and multifactorial disease affecting many interlinked pathological pathways. The lack of efficacy seen with the single target approach is compelling evidence that we needed to rethink the paradigm of drug design to treat these multifactorial diseases. MTD design offers a promising avenue for the progression of AD therapeutic intervention and could ultimately lead to a drug with the ability to reverse disease progression.

While there are many targets for disease-modifying drugs, it remains to be seen which combinations will prove efficacious. It seems logical that in order to reverse disease progression, we must target those aspects of the disease that cause neuronal damage, such as Aβ and oxidative damage, rather than just targeting the breakdown enzymes to alleviate the deficiencies caused by cell death. Of course, targeting a combination of both would relieve symptoms and prevent further neuronal loss.

In terms of the approach to MTD design, an integrated approach using a combination of *in silico* and rational drug design should reduce the cost of high throughput screening and progress discovery more rapidly. The MTD approach is also being used to combat the development of resistance to antimicrobials. MBX-500 has been designed as a hybrid of two classes of antibiotics, fluoroquinolone and anilinouracil, for the treatment of *Clostridium difficile* infections (Butler et al., 2012). TD-1792, a cephalosporin-vancomycin hybrid, has passed a phase II clinical trial for gram-positive skin infections (Stryjewski et al., 2012). While MTD design is in its infancy in the world of antibiotics, there is evidence that it could prove promising here too (East and Silver, 2013; Oldfield and Feng, 2014).

Overall, MTD design is a promising approach to modern medicines for complex diseases. Whether drugs will come from repurposing, rational drug design, *in silico* screening, or a combination approach we cannot predict, but the race is on to develop the first approved MTD capable of reversing AD pathology.

## Author contributions

REH reviewed the literature and wrote the draft. All authors contributed to the conception, interpretation, and critical revision of the work. All authors have approved the final version of this review of current literature.

## Funding

The authors thank COST Action CM1103 for the productive collaborations that inspired this work and for open access funding.

### Conflict of interest statement

The authors declare that the research was conducted in the absence of any commercial or financial relationships that could be construed as a potential conflict of interest.

## Abbreviations

AD: Alzheimer's Disease
AChE: acetylcholinesterase
Aβ: amyloid beta
MAO: monoamine oxidase
MTD: multi-target drug
N-methyl-D-aspartate: (NMDA receptor
GABA: gamma-aminobutyric acid
5-HT_4_R: serotonin (5-HT) receptor type 4
Glucagon-like peptide 1: (GLP-1)
BBB: blood brain barrier.
